# A Study of Structure and Magnetic Properties of Low Purity Fe-Co-Based Metallic Glasses

**DOI:** 10.3390/ma10060625

**Published:** 2017-06-08

**Authors:** Sabina Lesz

**Affiliations:** Faculty of Mechanical Engineering, Silesian University of Technology, 18a Konarskiego Street, Gliwice 44-100, Poland; sabina.lesz@polsl.pl; Tel.: +48-32-237-1577

**Keywords:** bulk metallic glasses (BMGs), conventional metallic glasses (CMGs), ferroalloys, XRD-method, HRTEM-method, magnetic properties

## Abstract

This paper is related to the evaluation of the possibility of using ferroalloys for the production of conventional (CMGs) and bulk metallic glasses (BMGs) as well as determining their magnetic properties. The structure and magnetic properties of Fe-Co-based CMGs and BMGs prepared from ferroalloys and pure elements, were studied. The CMGs and BMGs were in the form of ribbons and rods, respectively. The thickness of the ribbons were 0.07, 0.12, and 0.27 mm and the diameters of the rods were 1.5 and 2.5 mm. The investigations of the structure of the test specimens were carried out using the X-ray diffraction (XRD) method and electron microscopy methods (HRTEM—high-resolution transmission electron microscope, SEM—scanning electron microscope). The relationship between the structure and magnetic properties of the Fe_36.00_Co_36.00_B_19.00_Si_5_Nb_4_ and Fe_35.75_Co_35.75_B_18.90_Si_5_Nb_4_Cu_0.6_ CMGs and BMGs was determined. The possibility of using new materials, i.e., CMGs and BMGs, prepared on the basis of ferroalloys, lies in the scope of the presently conducted research and allows us to obtain the utility properties, while avoiding high costs associated with the purchase of raw materials.

## 1. Introduction

The Fe-Co-based conventional (CMGs) and bulk metallic glasses (BMGs) constitute potential research materials for institutions and industry because of their excellent GFA–glass forming ability, thermal stability, superior magnetic properties (high saturation magnetization *B*_s_, low coercivity *H*_c_) and good strength [1,2,3,4,5,6,7,8,9,10,11,12,13,14,15,16]. So far, these alloys have been prepared mainly from high purity elements (greater than 99.5 wt %). These metallic glasses can achieve a larger critical diameter than low purity metallic glasses (e.g., based on the ferroalloys). However, amorphous alloys, prepared from high purity metals and metalloids are very expensive because of raw materials’ purchase costs. Recently, the production of Fe-based metallic glasses (MGs) has been significantly increasing to improve energy saving needed for conservation of the environment, an urgent subject of the present times [17]. Because of the low core losses attributable to the good magnetic softness, energy saving is feasible. However, the thickness of the typical Fe-Si-B amorphous alloy ribbon produced by a melt-spinning method, in commercial production for many kinds of highly efficient transformers, is about 25 μm, which is much thinner than the other ordinary crystalline soft magnetic alloy sheets [1,18]. An Fe_84.2_Si_5.8_ crystalline alloy (silicon steel) sheet produced by cold rolling has some advantages, like unlimited thickness, high saturation magnetization (*B*_s_ ~ 2 T), and very low material cost. However, the silicon steel has a great disadvantage of inferior magnetic softness fundamentally caused by a large magneto-crystalline anisotropy and its production is long and expensive.

Fe- and Co-based MGs have the advantage over silicon steel, because their core loss is about 85% lower. This would therefore reduce the cost of operating electrical appliances. When comparing the Fe-based amorphous alloy ribbon and the silicon steel sheet, the production of a large thickness ribbon without decreasing (*B*_s_) and without increasing the material cost should be the first priority in the research of ferromagnetic amorphous alloys [17].

So far, lower power losses have been measured for the Fe-B-Si ribbons, than for the rods of the same composition, with a diameter of 0.8 mm. As a consequence, at this stage applications of these systems as power cores in transformers are not feasible. Further improvement can be obtained by tailoring the magnetic properties by changing the composition and/or geometry (i.e., toroidal (ring or donut) shape), exploiting the wide possibilities that bulk metallic glasses display. In fact, in the light of these results it has still to be taken into account the advantages offered by these systems as the one-step preparation together with the possibility of miniaturization for magnetic cores or inductive components [19]. The Fe-based CMGs and BMGs, because of their properties, have received considerable attention for use as core materials in magnetic heat, high-frequency, and high-power transformers, choke coils, magnetostrictive converters, and sensors [20]. The main users of cores are electronic equipment producers, especially manufacturers specializing in telecommunication and power supply equipment, etc. The amorphous and crystalline Fe-Co-based alloy has the potential for applications that require soft magnetic properties and thermal stability at temperatures as high as 900 °C [9,21].

The addition of alloying elements can affect the GFA, thermodynamical parameters, and structure, and thus determine the properties of metallic glasses. The coexistence of high saturation magnetization (*B*_s_) and high GFA is rare and this matter should be immensely important to all, from academia to industry in this material field [17].

The investigated alloys were based on ferromagnetic elements, which are: iron and cobalt which stand for concentrations at about 72 at %, ensuring high saturation magnetization. Other elements, i.e., boron and niobium, facilitate the formation of amorphous structures. Niobium, belonging to the vanadium group, plays a crucial role in the formation of the amorphous structure in the preparation process of ribbons [22]. In the casting process of amorphous ribbons from the liquid state, Nb has an effect on the GFA.

Silicon is the second most abundant element in the Earth’s crust. It is widely used in electronics and photovoltaics [23,24]. It is the significant addition, which has an effect on the GFA and thermodynamic parameters of alloys [25]. It is well known that increasing Si content in the Fe-Cu-Nb-Si-B and Fe-Si-B alloy systems can increase enthalpy of the first stage of crystallization and decrease the enthalpy of the second stage of crystallization [26].

Minor addition of Cu decreases the thermal stability of the supercooled liquid in the metallic glasses. The crystallization temperatures (*T*_x1_, *T*_x2_) are shifted to lower temperatures with the addition of Cu, which has the effect of raising the temperature interval of the supercooled liquid region Δ*T*_x_. The presence of Cu, which insignificantly solubilizes in iron, leads to forming Cu-rich clusters prior to the onset of crystallization. Cu influences the type of α Fe(Si) phase, formed during the first stage of crystallization in the MGs containing Nb and thus effectively affects the magnetic properties [27]. Results presented by Yoshizawa [28] and Müller [14] show that he optimum concentration of the Cu element for obtaining the best soft magnetic properties is 1 at % in Fe-Nb-Si-B-Cu alloys, 0.5 at % in Fe-C-Si-B-P-Cu alloys [29], and 0.6 at % in Fe-Co-B-Si-Nb-Cu alloys [2,3]. Thus, insignificant modification of the chemical composition by Cu addition allows us to achieve good soft magnetic properties.

The manufacturing technology of crystalline metals is well developed and commercialized [30,31,32], while the manufacturing technology of BMGs has not been well established and available to the same extent yet. Different casting and softening methods and mainly high purity charge materials are used for manufacturing BMGs. BMG properties depend on the cooling rate, so it is essential to choose the appropriate manufacturing method and define parameters of the casting process using the methods of rapid alloy cooling from the liquid phase, in particular with reference to the pressure die-casting into a copper mold. Previous research and technological experience in this area and the market application prospects indicate the need for further development of the research on metallic glass production technologies, while fulfilling the criteria for the minimization of the energy and material consumption. The unrelenting pressure on cost reduction makes the economic factor crucial in the selection of engineering materials. It is reflected in striving for the reduction of material costs, for example by replacing pure elements with charged materials in the form of lower purity metals and alloys, such as ferroalloys, in the production of CMGs and BMGs.

However, for these alloys there are difficulties in achieving a fully amorphous structure due to the presence of impurities. Impurities may form crystal nuclei and cause problems with obtaining the eutectic composition. Thus, solving these problems becomes an issue of interesting and innovative scientific works. Available in the literature [1,3,8,9,13,14,17,25], the results of investigations of the structure and properties of metallic glasses are mainly based on alloys produced from pure elements. Although the metallic glasses have been manufactured and investigated for many years, the current knowledge about the structure and properties of MGs prepared on the basis of industrial materials, such as ferroalloys, remains limited [33,34,35,36]. Of the available ferroalloys, Fe-B is mainly used for manufacturing metallic glasses [33,34,35,36]. In the literature, there are still no data on the structure and properties of the metallic glasses made by combining a few ferroalloys (Fe-B, Fe-Si, Fe-Nb), which determine their industrial application [37]. Another issue that has not been presented in a comprehensive way so far is the analysis of structural components in the structure of partially amorphous Fe-Co-B-Si-Nb metallic glasses. A crucial importance for their application is to establish a relationship between the presence of crystalline boride phases and the maintenance of the magnetic properties of Fe-based BMGs.

## 2. Materials and Methods

### 2.1. Materials

The Fe_36.00_Co_36.00_B_19.00_Si_5_Nb_4_ and Fe_35.75_Co_35.75_B_18.90_Si_5_Nb_4_Cu_0.6_ master alloy ingots were prepared by melting the mixtures of the following ferroalloys: Fe-B (Fe 85.4 B 14.5 wt %), Fe-Nb (Fe 31.4 Nb 68.5 wt %), and Fe-Si (Fe 42.7 Si 57.2 wt %). Ferroalloys belong to the alloys with less than 50 wt % content of iron and with one or more other elements, e.g., boron, silicon. These contain besides the main elements, like iron and boron, other constitutes such as aluminium (0.2–1.5 wt %), silicon (0.3–3.0 wt %), carbon (0.1–0.5 wt %), sulphur (0.015–0.1 wt %), and phosphorus (0.02–0.1 wt %). They are used as raw materials for steel manufacturing. The ferroalloys applied in this work were obtained from industrial large-scale production. The content of boron, niobium, and silicon in the cast alloy were adjusted by adding the Fe-B, Fe-Nb, and Fe-Si alloys, respectively, which are much cheaper than pure elements. Pure elements, such as Fe (99.99 wt %), Co (99.99 wt %), and Cu (99.99 wt %) were added to achieve the chemical composition of the alloys. The ingots were re-melted a few times in a furnace with purified argon atmosphere to ensure their homogeneity, and the Fe_36.00_Co_36.00_B_19.00_Si_5_Nb_4_ and Fe_35.75_Co_35.75_B_18.90_Si_5_Nb_4_Cu_0.6_ alloy compositions represent nominal atomic percentages. Ribbons with the thicknesses of 0.07, 0.12, and 0.27 mm and width of 2.3 mm were produced by the single copper roller melt spinning method. The master alloy was melted in a quartz crucible using an induction coil and pushed thereafter on a copper wheel by applying an ejection pressure of about 0.02 MPa. Bulk samples in rod form with the diameters of 1.5 and 2.5 mm were prepared by the pressure die casting method into a copper mold.

### 2.2. Methods

The microstructure of the samples (ribbons and rods) was examined by the X-ray diffraction (XRD) method and high-resolution transmission electron microscopy (HRTEM, FEI, Eindhoven, The Netherlands). XRD measurements were performed at ambient temperature using a X-Pert PRO MP diffractometer (PANalytical, Almelo, The Netherlands) with Co-Kα radiation (λ = 0.17888 nm), a tube voltage of 30 kV, and a current of 10  mA, in Bragg-Brentano geometry. Phase qualitative analysis was performed using classical X-ray diffraction. X-ray Diffraction (XRD) can be used to determine phase composition (commonly called phase ID) for mixtures of crystalline and nanocrystalline metals, foils, coatings, films, etc. [38]. The result of the analysis consists of an identified phase list with experimentally observed X-ray patterns and known diffraction patterns from various sources. The sources and the notation describing the quality of data are maintained by the ICDD (International Centre for Diffraction Data) [39]. The peaks were compared to probable crystalline species using the software X’Pert Highscore Plus 3.0e correlated with PAN-ICSD data base (2013-1, PANalytical, Almelo, The Netherlands) The structures of ribbons and rods were also examined by a high-resolution transmission electron microscope (HRTEM) S/TEM TITAN 80–300 FEI. Structures were observed on the small lamellas. The lamella-formed samples of 20 μm × 8 μm were cut out with a gallium-focused ion beam from a specific cross-sectional area of the test specimen and were then thinned with this ion beam to the thickness of approximately 50–70 nm using a FEI FIB Quanta 3D200 machine (FEI, Eindhoven, The Netherlands) [40]. The lamella-preparation process was observed in situ using a scanning electron microscope.

The morphology of fracture surfaces of the samples after decohesion was examined by means of a scanning electron microscope (SEM) SUPRA 35, with a voltage of 20 kV (Carl Zeiss, Jena, Germany). Analysis of the chemical composition of the samples was performed using an EDAX energy-dispersive X-ray spectroscope (EDS), coupled with the SEM. The boron content in the alloy composition was not determined.

A vibrating sample magnetometer (VSM), (LakeShore, Euclid, OH, USA), was used to measure the magnetic properties of the samples. High field magnetization curves were measured using magnetic induction up to 2 T. The magnetizing field was parallel to the sample length to minimize the demagnetization effect. The magnetization curves were analyzed using the least squares method. The initial magnetic permeability *μ_i_* of the ribbon samples was measured by using the Maxwell-Wien bridge (Agilent HP, Tokyo, Japan). The applied magnetic field had a value of 0.5 A/m and a frequency of about 1 kHz. The magnetic after effects (*Δμ/μ*) were determined by measuring changes of magnetic permeability of the examined alloys as a function of time after demagnetization, where *∆µ* is the difference in magnetic permeability measured at *t*_1_ = 30 s and *t*_2_ = 1800 s after demagnetization [41,42].

## 3. Results and Discussion

**Figure 1:**
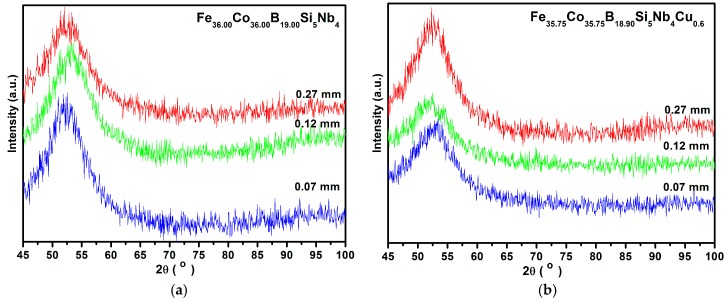
X-ray diffraction patterns of (**a**) Fe_36.00_Co_36.00_B_19.00_Si_5_Nb_4_ and (**b**) Fe_35.75_Co_35.75_B_18.90_Si_5_Nb_4_Cu_0.6_ alloys in ribbon form with thickness of 0.07, 0.12, and 0.27 mm.

**Figure 2:**
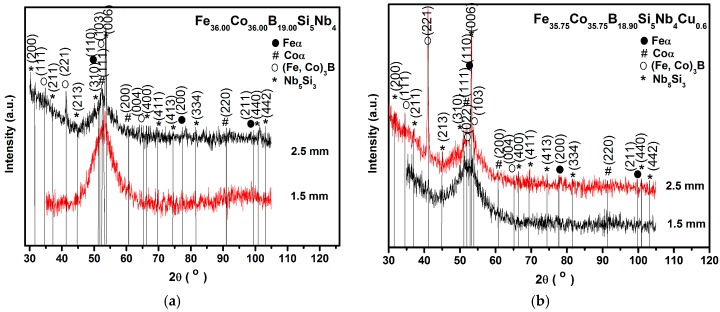
X-ray diffraction patterns of (**a**) Fe_36.00_Co_36.00_B_19.00_Si_5_Nb_4_ and (**b**) Fe_35.75_Co_35.75_B_18.90_Si_5_Nb_4_Cu_0.6_ alloys in rod form with diameters of 1.5 and 2.5 mm.

**Figure 3:**
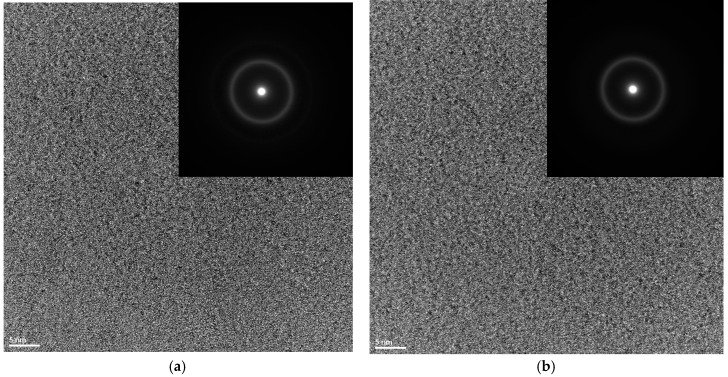
High-resolution transmission electron microscopy (HRTEM) images and the selected area electron diffractions (SAED) of (**a**) Fe_36.00_Co_36.00_B_19.00_Si_5_Nb_4_ and (**b**) Fe_35.75_Co_35.75_B_18.90_Si_5_Nb_4_Cu_0.6_ amorphous alloys in ribbon form with the thickness of 0.07 mm.

**Figure 4:**
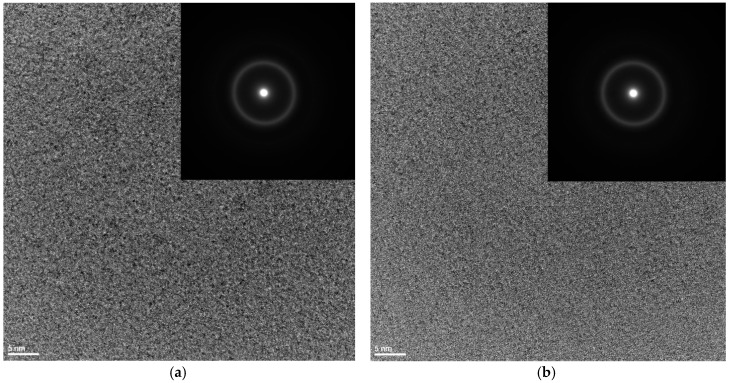
High-resolution transmission electron microscopy (HTREM) images and the selected area electron diffraction (SAED) of (**a**) Fe_36.00_Co_36.00_B_19.00_Si_5_Nb_4_ and (**b**) Fe_35.75_Co_35.75_B_18.90_Si_5_Nb_4_Cu_0.6_ amorphous alloys in rod form with the diameter of 1.5 mm.

**Figure 5:**
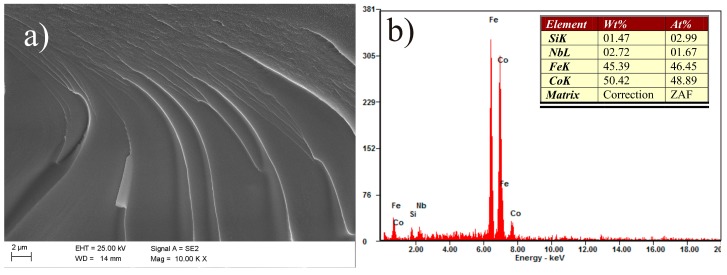
Scanning electron microscopy (SEM) micrographs of the fracture morphology of Fe_36.00_Co_36.00_B_19.00_Si_5_Nb_4_ ribbon with the thickness of 0.07 mm: (**a**) view; (**b**) Energy-dispersive X-ray spectroscope (EDS) spectrum from area in (**a**).

**Figure 6:**
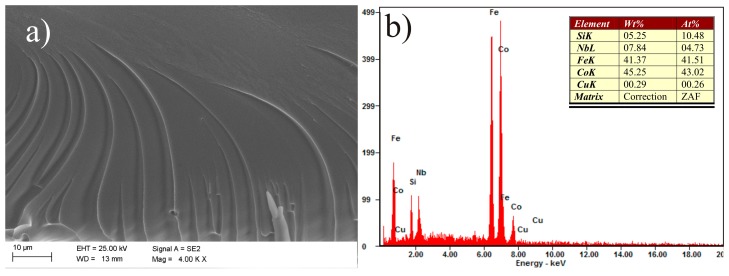
SEM micrographs of the fracture morphology of Fe_35.75_Co_35.75_B_18.90_Si_5_Nb_4_Cu_0.6_ ribbon with the thickness of 0.07 mm: (**a**) view; (**b**) EDS spectrum from area in (a).

The results of the investigations of the structure of ribbons with different thicknesses produced from Fe_36.00_Co_36.00_B_19.00_Si_5_Nb_4_ and Fe_35.75_Co_35.75_B_18.90_Si_5_Nb_4_Cu_0,6_ alloys carried out by the X-ray phase analysis method have shown that the ribbons are in the amorphous state (Figure 1a,b). The structure of the rods with the diameter of 1.5 mm is mainly amorphous (Figure 2a,b). In Figure 2a,b only one pattern respectively has a broad-angle peak. This is evidenced by numerous structural images taken by the latest techniques of high-resolution transmission electron microscopy (HRTEM) (Figure 3a,b and Figure 4a,b) and scanning electron microscopy (SEM) (Figure 5 and Figure 6).

Figure 1a,b shows XRD patterns of the investigated alloy in ribbon form with the thicknesses of 0.07, 0.12, and 0.27 mm. Typical diffused scatterings indicate the presence of a mainly amorphous structure with no crystalline peaks.

The obtained results of structural studies of ribbons and the rod with the diameter of 1.5 mm performed by the HRTEM micrograph are presented in Figure 3 and Figure 4. An example of the HRTEM images of the ribbon samples with the thicknesses of 0.07, 0.12, and 0.27 mm is shown in Figure 3a. Figure 4a presents the HRTEM image of the rod with the diameter of 1.5 mm. The selected area electron diffractions (SAED) taken from these samples are shown in Figure 3b and Figure 4b. In the images (Figure 3a and Figure 4a), we can observe the positions with randomly distributed dark and bright contrasts. From the HRTEM images we may conclude that local short-range order (SRO) regions have occurred during the cooling of the Fe-Co-based metallic glass. The selected area electron diffraction (SAED) pattern consisted only of halo rings (Figure 3b and Figure 4b). The diffuse diffraction halos may be taken as characteristic of the amorphous state. However, this characteristic alone is not sufficient to describe the atomic arrangements within the solid. Although the precise description of atomic structures for metallic glasses is still open even now.

Vein pattern morphology was observed in the investigated ribbons, typical of amorphous alloys [43]. The morphology is changing from a smooth fracture inside with a few vein networks in the surface freely solidified (shining surface) (Figure 5a and Figure 6a). Macroscopically metallic glasses behave in a brittle manner. Microanalysis of the chemical composition in the EDS plots, examined by SEM, is shown in Figure 5b and Figure 6b and confirms that the Fe_36.00_Co_36.00_B_19.00_Si_5_Nb_4_ and Fe_35.75_Co_35.75_B_18.90_Si_5_Nb_4_Cu_0.__6_ ribbons are composed of the alloyed elements Fe, Co, Si, Nb, and Cu.

**Figure 7:**
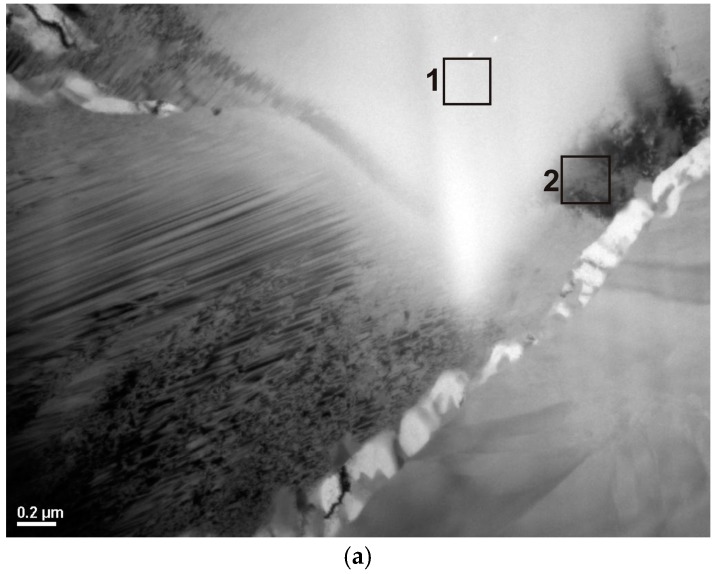

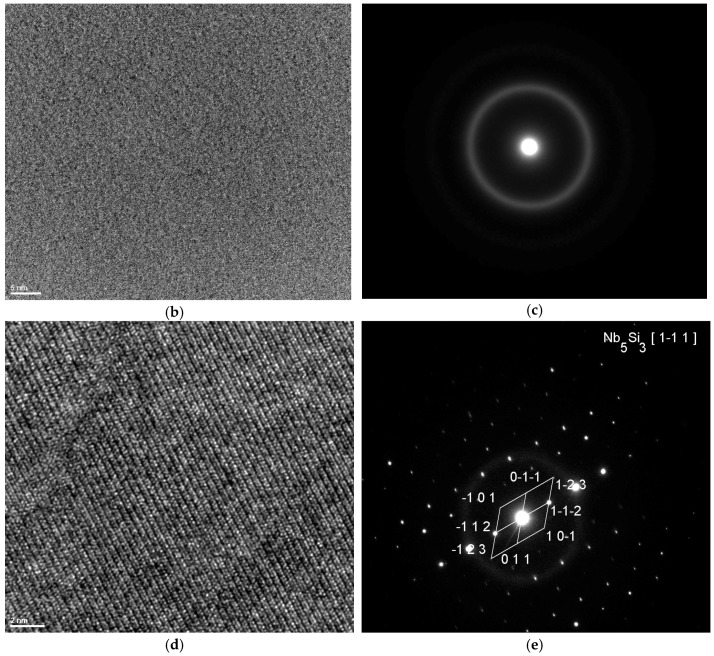
Transmission electron microscopy (TEM) images of the Fe_36.00_Co_36.00_B_19.00_Si_5_Nb_4_ alloy in rod form with the diameter of 2.5 mm: (**a**) bright field; (**b**) HRTEM image of the position marked 1 in (a) at higher magnification; (**c**) electron diffraction, an amorphous phase; (**d**) HRTEM image of the position marked 2 in (a) at higher magnification; (**e**) electron diffraction with solution, precipitate of the Nb_5_Si_3_ phase.

**Figure 8:**
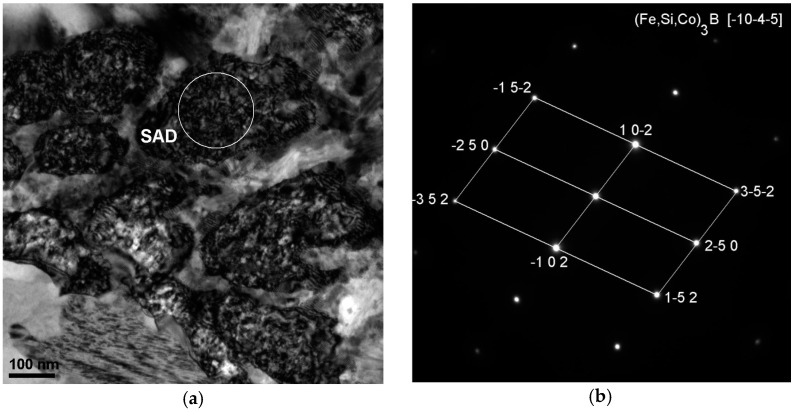
TEM image of the Fe_36.00_Co_36.00_B_19.00_Si_5_Nb_4_ alloy in rod form with the diameter of 2.5 mm, (**a**) bright field; (**b**) the electron diffraction from the position marked SAD in (a); solution of diffraction: (Fe,Si,Co)_3_B phase.

**Figure 9:**
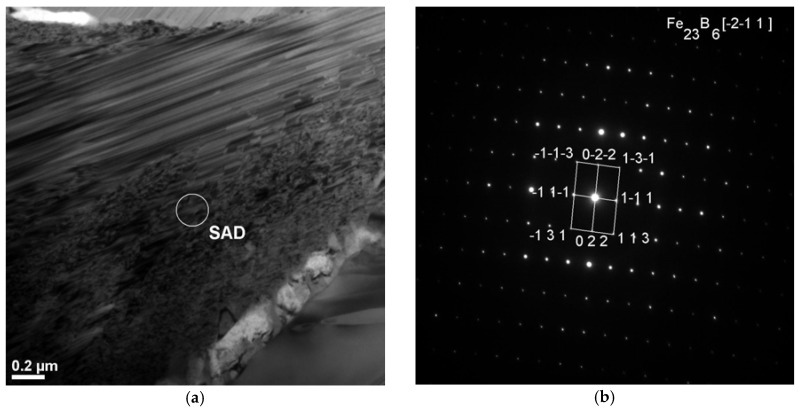
TEM image of the Fe_36.00_Co_36.00_B_19.00_Si_5_Nb_4_ alloy in rod form with the diameter of 2.5 mm, (**a**) bright field; (**b**) the electron diffraction from the position marked SAD in (a); solution of diffraction: Fe_23_B_6_ phase.

**Figure 10:**
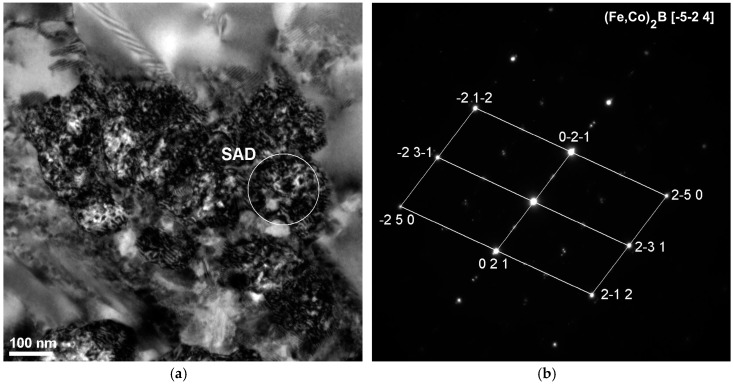
TEM image of the Fe_36.00_Co_36.00_B_19.00_Si_5_Nb_4_Fe_35.75_Co_35.75_B_18.90_Si_5_Nb_4_Cu_0.6_ alloy in rod form with the diameter of 2.5 mm, (**a**) bright field; (**b**) the electron diffraction from the position marked SAD in (a); solution of diffraction: (Fe,Co)B_2_ phase.

However, rods with the diameter of 2.5 mm are partially crystallized (in Figure 2a,b only one pattern). The results of the X-ray diffraction method using the Fe_36.00_Co_36.00_B_19.00_Si_5_Nb_4_ and Fe_35.75_Co_35.75_B_18.90_Si_5_Nb_4_Cu_0.6_ rods with the diameter of 2.5 mm indicates that their phase composition is the same (Figure 2a,b). In the XRD patterns, a few of the Bragg’s peaks are superimposed on the diffused diffraction maxima, which means that the rods contain crystalline phases. The crystalline phases are identified as (Fe, Co)α, (Fe, Co, Si)_3_B, and Nb_5_Si_3_ [39]. The results of the HRTEM confirm the existence of the amorphous (Figure 7a–c) and crystalline phases (Figure 7a,d,e, Figure 8a,b, Figure 9a,b and Figure 10a,b) in the structure of the rods.

The existence of the crystalline phases Fe_23_B_6_ and Fe_3_B and Nb-rich phase have been reported in the Fe_77−*x*_Co*_x_*Nb_7_B_15_Cu_1_ (*x* = 0, 2, 4, 8 at %) alloy [43]. The metastable iron boride Fe_23_B_6_ phase was confirmed by the XRD (X-Ray Diffraction) and D (Differential Scanning Calorimetry) results achieved for the Fe-Co-Si-B-Nb alloy prepared with the high purity metals [16,44]. The metastable Fe_3_B phase is favored in the alloy with the replacement of Fe by Co [44]. Comparing with the DSC measured for the other [(Fe_0.5_Co_0.5_)_0.75_B_0.20_Si_0.05_]_96_Nb_4_ alloy, the middle of the second crystallization peak and some small additional peaks can be seen and they could be identified as (Fe,Co)_3_B or (Fe,Co)_2_B [45]. Most probably, the crystallization sequence of the [(Fe_0.5_Co_0.5_)_0.75_B_0.20_Si_0.05_]_96_Nb_4_ alloy is as follows: amorphous→ Fe_23_B_6_-type plus residual amorphous→ Fe_23_B_6_-type plus Fe_2_B or Fe_3_B plus residual amorphous→ Fe_23_B_6_ plus Fe_2_B or Fe_3_B [45].

For the (Fe_36_Co_36_B_19.2_Si_4.8_Nb_4_)_100−*x*_Cu*_x_* alloy, this sequence is different and can be summarized as follows: amorphous phase 1→ amorphous phase 2 plus (Fe,Co)→ residual amorphous phase (eventually) plus (Fe,Co) plus (Fe,Co,Nb)_23_B_6_→ (Fe,Co) plus (Fe,Co,Nb)_23_B_6_ plus (Fe,Co)_2_B [46]. These differences in the crystallization behavior are associated with the different atomic arrangements in the two types of amorphous alloys. This hypothesis was confirmed by synchrotron studies [45,46]. For the {[(Fe_0.5_Co_0.5_)_0.75_B_0.20_Si_0.05_]_0.96_Nb_0_._04_}_100−*x*_Cu*_x_* alloy prepared on the basis of pure metals and for the (Fe_36_Co_36_B_19,2_Si_4,8_Nb_4_)_100−*x*_Cu*_x_* and [(Fe,Co)_0,75_Si_0,05_B_0,20_]_94_Nb_6_ alloys prepared on the ferroalloys, the existence of the crystalline phases, such as (Fe,Co)_23_B_6_, (Fe,Co)_2_B, (Fe,Co)_3_B, and Nb_5_Si_3_ was previously shown in the literature [4,5,46].

The analysis of structural components in the structure of partially crystallized amorphous Fe-Co-B-Si-Nb rods with the diameter of 2.5 mm carried out on the basis of the results of the X-ray examinations (Figure 2a,b) and transmission electron microscopy (TEM) (Figure 7, Figure 8, Figure 9 and Figure 10) allowed us to establish the relationship between the presence of crystalline boride phases and the maintenance of magnetic properties. These results have significant informative and application importance.

The ability to obtain the amorphous state for the rods with the diameter of 2.5 mm is highly affected by the purity of the charged materials that the test specimens are made from. If pure metals and non-metals are used, the rods with the diameter of 2.5 mm are also obtained in the amorphous state [47,48].

**Figure 11:**
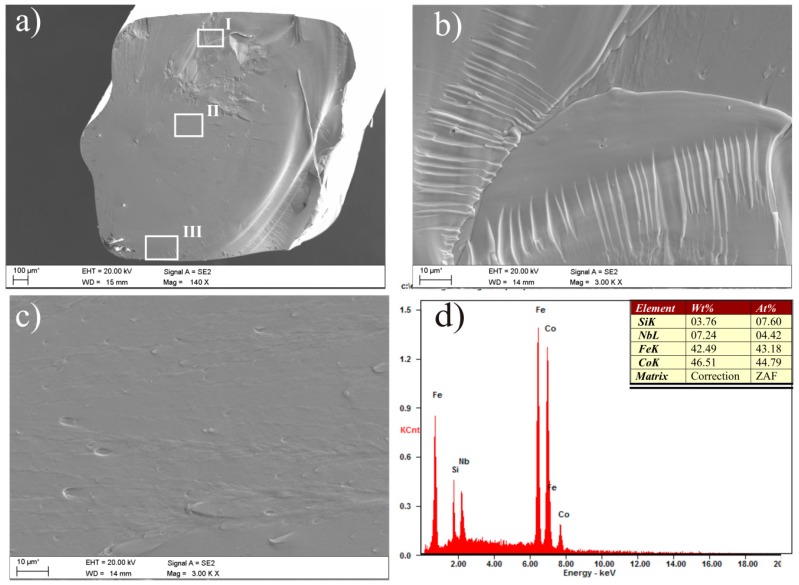

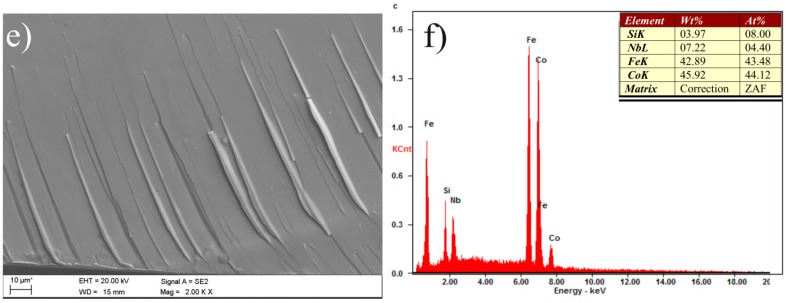
SEM micrographs of the fracture morphology of the Fe_36.00_Co_36.00_B_19.00_Si_5_Nb_4_ rod with the diameter of 1.5 mm; (**a**) main view; (**b**) image of fragment I marked in (a) at higher magnification; (**c**) image of fragment II marked in (a) at higher magnification; (**d**) EDS spectrum from area in (c); (**e**) image of fragment III marked in (a)) at higher magnification; (**f**) EDS spectrum from area in (e).

**Figure 12:**
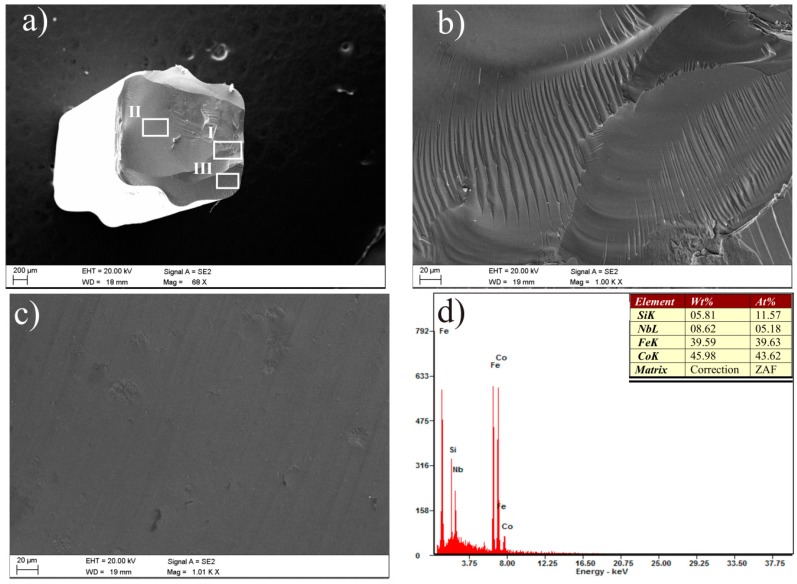

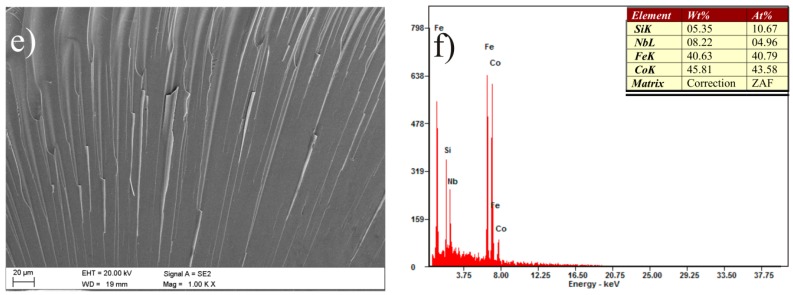
SEM micrographs of the fracture morphology of the Fe_35.75_Co_35.75_B_18.90_Si_5_Nb_4_Cu_0.6_ rod with the diameter of 1.5 mm; (**a**) main view; (**b**) image of fragment I marked in (a) at higher magnification; (**c**) image of fragment II marked in (a) at higher magnification; (**d**) EDS spectrum from area in (c); (**e**) image of fragment III marked in (a) at higher magnification; (**f**) EDS spectrum from area in (e).

The fracture morphology using the Fe_36.00_Co_36.00_B_19.00_Si_5_Nb_4_ and Fe_35.75_Co_35.75_B_18.90_Si_5_Nb_4_Cu_0.6_ rods was different in the cross sections. This depends on the diameter of the rods and the space analysis (Figure 11 and Figure 12). These fracture surfaces of the rods with the diameters of 1.5 and 2.5 mm are typical for the relaxation of metallic glasses [49,50]. Smooth fracture morphology with areas of vein and chevron pattern morphology have been observed in the region between the surface and the core of the rod and in the region of direct contact of the liquid alloy in a copper mold. A smooth pattern is usually observed at the fracture surface of brittle MGs [43].

**Figure 13:**
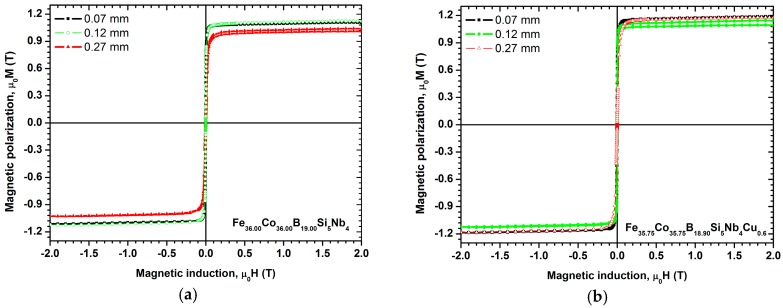
Room temperature magnetic hysteresis loops measured at a maximum magnetic induction of *μ_0_H* = 2 T for samples of (**a**) Fe_36.00_Co_36.00_B_19.00_Si_5_Nb_4_ and (**b**) Fe_35.75_Co_35.75_B_18.90_Si_5_Nb_4_Cu_0.6_ alloys in ribbon form with thicknesses of 0.07, 0.12, and 0.27 mm.

**Figure 14:**
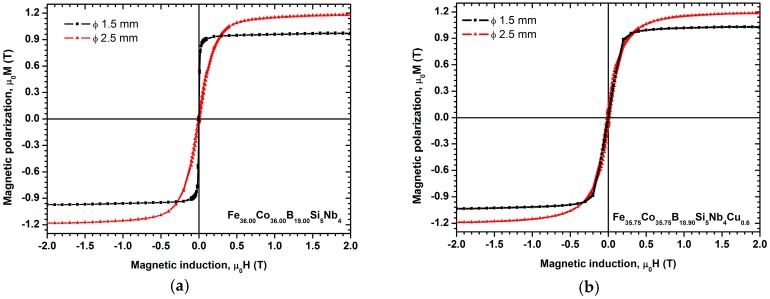
Room temperature magnetic hysteresis loops measured at a maximum magnetic induction of *μ_0_H* = 2 T for samples of (**a**) Fe_36.00_Co_36.00_B_19.00_Si_5_Nb_4_ and (**b**) Fe_35.75_Co_35.75_B_18.90_Si_5_Nb_4_Cu_0.6_ alloys in rod form with diameters of 1.5 and 2.5 mm.

The results of the magnetic properties studies of the test specimens from the Fe_36.00_Co_36.00_B_19.00_Si_5_Nb_4_ and the Fe_35.75_Co_35.75_B_18.90_Si_5_Nb_4_Cu_0.6_ alloys are presented in Table 1. The results of the magnetic properties (the saturation magnetic polarization—*J*_s_ and coercivity—*H*_c_) obtained with a vibrating sample magnetometer (VSM) show the hysteresis loops, shown in Figure 13 and Figure 14.

The coercive force *H*_c_ decreases from the value of 65.2 A/m using the Fe_36.00_Co_36.00_B_19.00_Si_5_Nb_4_ alloy in the form of ribbons with the thickness of 0.07 mm to the value of 53.5 A/m using the Fe_35.75_Co_35.75_B_18.90_Si_5_Nb_4_Cu_0.6_ alloy (Figure 13a,b). A similar decrease of the coercive force *H*_c_ was observed using the Fe_36.00_Co_36.00_B_19.00_Si_5_Nb_4_ and Fe_35.75_Co_35.75_B_18.90_Si_5_Nb_4_Cu_0.6_ alloys in the form of ribbons with the thicknesses of 0.12 (Figure 13a,b) and 0.27 mm (Figure 13a,b).

The investigated ribbons of alloys retain their ferromagnetic properties at room temperature, showing low coercive force *H*_c_ and high saturation magnetic polarization *J*_s_ (Table 1, Figure 13a,b). The highest saturation magnetic polarization *J*_s_ = 1.18 T and relative initial magnetic permeability are demonstrated by the Fe_35.75_Co_35.75_B_18.90_Si_5_Nb_4_Cu_0.6_ alloy in the form of ribbons with the thicknesses of 0.07 and 0.27 mm (Table 1).

For alloy rods containing copper, the saturation magnetic polarization *J*_s_ was from 1.03 to 1.18 T (Figure 14b), which is similar to that of the base Fe_36.00_Co_36.00_B_19.00_Si_5_Nb_4_ alloy—*J*_s_ was from 0.97 to 1.17 T (Figure 14a). It has been reported that the addition of copper into Fe-Co-B-Si-Nb alloys improves the soft magnetic properties of the alloy, by decreasing the coercive force and increasing the relative initial magnetic permeability—*μ_i_*. This is probably connected with the impurity content. Jia reported that the Cu addition reduces the oxygen content of the alloy [2].

The alloy rods prepared on the basis of pure metals and non-metals have a lower coercive force [45,46,47] compared to the alloy rods prepared on the basis of ferroalloys, which is related to the reduced amount of impurities present in the alloy [2]. The use of high-purity charge materials makes it possible to obtain an increased critical casting thickness/diameter of BMGs [47,48]. The critical diameter of BMGs is the maximal dimension of the sample having an amorphous structure. The highest coercive force is observed in the rods with the diameter of 2.5 mm prepared on the basis of ferroalloys that can be ascribed to the appearance of boride phases in the structure of the alloy.

In the amorphous alloy, the domain walls are free to move due to the low magnetocrystalline anisotropy [26], resulting in a low *H*_c_*.* The lower coercivity could be obtained upon annealing well below *T*_x_ in order to reduce the stress field in the as-cast amorphous matrix or structural relaxation phenomena. A relaxed amorphous phase in the material produced at lower rates of cooling is characterized by the presence of the local atomic order and magnetic structure (different than the nano-structure). Cu atom clusters are present in the amorphous structure of the Fe-Co-Si-Nb-B alloy containing Cu. The Cu atoms aggregate to form small clusters and the size of the clusters is approximately a few nm. [51,52]. Improvement of the soft magnetic properties of the alloy is caused by structural relaxation. However, when there are impurities in the alloy, they can pin the domain walls [26]. This might result in a high coercive force—*H*_c_ [53,54].

Ribbons with lower thickness show higher magnetic permeability relaxation intensity after demagnetization (*Δμ/μ*) than those with higher thickness. The values of *Δμ/μ* were 5, 3.6, and 3.0% using the Fe_36.00_Co_36.00_B_19.00_Si_5_Nb_4_ alloy in the form of ribbons with the thicknesses of 0.07, 0.12, and 0.27 mm, respectively. In turn, higher values of *Δμ/μ* = 11, 10, and 8% were obtained using the Fe_35.75_Co_35.75_B_18.90_Si_5_Nb_4_Cu_0.6_ alloy in the form of ribbons with the thicknesss of 0.07, 0.12, and 0.27 mm, respectively (Table 1). The magnetic permeability relaxation intensity after demagnetization is proportional to the concentration of microvoids (excess volume) in the magnetic metallic glasses [10,41,42]. The share of microvoids in the alloy depends on the conditions of the casting process.

Cu addition in the Fe-Co-Nb-Si-B alloy makes the magnetic permeability relaxation intensity increase after demagnetization (*Δμ/μ*) as a result of the presence of free volumes. Because copper has a lower melting temperature than iron and cobalt, it causes an increase of the free volume in the system during solidification. The copper diffusion produces a chemical inhomogeneity of the alloying elements, particularly Fe, which is induced by the Cu-cluster formation and which in turn causes an increase of the number of nucleation sites for the formation of the crystalline phase. The Cu atoms start to form a crystalline phase prior to the formation of the nanocrystalline phase [51,52].

Thermal methods, such as differential thermal analysis (DTA) (NETZSCH, Selb, Germany) and differential scanning calorimetry (DSC) (NETZSCH, Selb, Germany), allowed the characteristic temperatures of the examined alloys to be determined, i.e., crystallization onset temperature (*T*_x1_), eutectic temperature (*T*_e_), melting point (*T*_m_), and glass forming ability (GFA) indicators (*T*_g_—glass transition temperature; *T*_l_—liquidus temperature; Δ*T*_x_ = *T*_x1_ − *T*_g_—range of supercooled liquid; *T*_rg_ = *T*_g_/*T*_l_—reduced glass transition temperature) [42,55]. The chemical composition of the examined alloys was similar to the eutectic one, which assures their good glass forming ability [42,55].

In addition to purely utilitarian features, the investigations conducted on the magnetic properties have also provided results that are useful for analysis of the structure of the amorphous state. Magnetic methods, such as initial permeability, coercive force, and magnetic relaxation intensity, depend on the state of stress in the material and the concentration of microvoids. The results of investigations obtained with VSM vibrating magnetometer and presented in the form of hysteresis loop allow the examined alloys to be classified as soft magnetic materials. The obtained values for relative initial permeability *μ_i_* and coercive force correlate with each other for ribbons and rods with the diameter of 1.5 mm.

## 4. Conclusions

The results of this research provided the grounds for drawing the following conclusions:

- Charge materials in the form of ferroalloys allow production of conventional and bulk metallic glasses with good soft magnetic properties, thus ensuring the reduction of manufacturing costs.
- Produced materials with amorphous structures, which are confirmed by direct methods, such as XRD, TEM, SEM-methods, and indirect methods, such as magnetic properties investigations, meet the most important requirements for soft magnetic materials at room temperature and show low coercive force *H*_c_ and high saturation magnetic polarization *J*_s_. The highest saturation magnetic polarization *J*_s_ = 1.18 T and relative initial magnetic permeability are demonstrated by the Fe_35.75_Co_35.75_B_18.90_Si_5_Nb_4_Cu_0.6_ alloy in the form of ribbons with the thicknesses of 0.07 and 0.27 mm. For alloy rods containing copper, the saturation magnetic polarization *J*_s_ was from 1.03 to 1.18 T, which is similar to that of the base Fe_36.00_Co_36.00_B_19.00_Si_5_Nb_4_ alloy—*J*_s_ was from 0.97 to 1.17 T. It is determined by the higher excess volume concentration for test specimens in the form of ribbons than for those in the form of rods, which is caused by the application of a higher cooling rate of ribbons.
- The casting process parameters affect the share of microvoids in the alloy, and consequently the obtained magnetic properties. Ribbons with lower thickness show higher magnetic permeability relaxation intensity after demagnetization (*Δμ/μ*) than those with higher thickness. The magnetic permeability relaxation intensity after demagnetization is proportional to the concentration of microvoids (excess volume) in magnetic metallic glasses. Cu addition in the Fe-Co-Nb-Si-B alloy makes the magnetic permeability relaxation intensity increase after demagnetization (*Δμ/μ*) as a result of the presence of free volumes and Cu clusters in the amorphous structure of the alloy, even before the actual crystallization process has started.
- Copper addition improves the soft magnetic properties of the alloy, by decreasing the coercive force and increasing the relative initial magnetic permeability—*μ_i_*. Lower coercive force is caused by structural relaxation, which allows the reduction in stress present in the amorphous matrix, or by the presence of Cu clusters in the alloys containing Cu. The presence of impurities in the alloy hinders the movement of domain walls, thus resulting in a high coercive force—*H*_c_.
- The highest coercive force is observed in rods with the diameter of 2.5 mm. The deterioration in magnetic softness is connected with the appearance of boride phases in the structure of the alloy, which are characterized by strong magnetocrystalline anisotropy.

## Figures and Tables

**Table 1:** The results of magnetic properties (*µ_i_*, *Δµ/µ*, *H*_c_, *J*_s_) of the test specimens from the Fe_36.00_Co_36.00_B_19.00_Si_5_Nb_4_ and the Fe_35.75_Co_35.75_B_18.90_Si_5_Nb_4_Cu_0.6_ alloys in the form of ribbons and rods.

Alloy	Thickness ^1^/	-	Magnetic	Properties	-
-	Diameters ^2^ (mm)	*μ_i_*	*Δµ/µ* (%)	*H*_c_ (A/m)	*J*_s_ (T)
Fe_36.00_Co_36.00_B_19.00_Si_5_Nb_4_	0.07 ^1^	3000	5.0	65.2	1.11
Fe_36.00_Co_36.00_B_19.00_Si_5_Nb_4_	0.12 ^1^	1567	3.6	41.4	1.12
Fe_36.00_Co_36.00_B_19.00_Si_5_Nb_4_	0.27 ^1^	410	3.0	30.7	1.04
Fe_36.00_Co_36.00_B_19.00_Si_5_Nb_4_	1.5 ^2^	-	-	219.0	0.97
Fe_36.00_Co_36.00_B_19.00_Si_5_Nb_4_	2.5 ^2^	-	-	6069.4	1.17
Fe_35.75_Co_35.75_B_18.90_Si_5_Nb_4_Cu_0.6_	0.07 ^1^	3620	11.0	53.5	1.18
Fe_35.75_Co_35.75_B_18.90_Si_5_Nb_4_Cu_0.6_	0.12 ^1^	1980	10.0	20.5	1.14
Fe_35.75_Co_35.75_B_18.90_Si_5_Nb_4_Cu_0.6_	0.27 ^1^	820	8.0	12.5	1.18
Fe_35.75_Co_35.75_B_18.90_Si_5_Nb_4_Cu_0.6_	1.5 ^2^	-	-	163.0	1.03
Fe_35.75_Co_35.75_B_18.90_Si_5_Nb_4_Cu_0.6_	2.5 ^2^	-	-	13771.8	1.18

^1^ alloys in ribbon form, ^2^ alloys in rod form.
